# Quantification of Enteric Dysfunction in Cystic Fibrosis: Inter- and Intraindividual Variability

**DOI:** 10.1016/j.jpeds.2023.113800

**Published:** 2024-02

**Authors:** Laura A. Duckworth, Kimberly A. Sutton, Nurmohammad Shaikh, Jinli Wang, Carla Hall-Moore, Lori R. Holtz, Phillip I. Tarr, Ronald C. Rubenstein

**Affiliations:** 1 Division of Gastroenterology, Hepatology, and Nutrition, Department of Pediatrics, Washington University in St Louis, St Louis, MO; 2 Center for Biostatistics and Data Science, Washington University in St Louis, St Louis, MO; 3 Division of Allergy and Pulmonary Medicine, Department of Pediatrics, Washington University in St Louis, St Louis, MO

**Keywords:** intestinal inflammation, intestinal permeability, fecal lipocalin-2, fecal neopterin, fecal calprotectin, plasma lipopolysaccharide antibody, plasma lipopolysaccharide-binding protein

## Abstract

**Objectives:**

To test the utility of various biomarkers as indicators of gut dysfunction in cystic fibrosis (CF) and determine whether intraindividual variations in these measures are repeatable over short intervals and whether interindividual variations correlate with clinical outcomes.

**Study design:**

We performed a cross-sectional, limited longitudinal study of children with CF aged 1-21 years who provided blood and stool samples at 2 or 3 visits, 2 weeks and 3 months apart, which were assayed for markers of intestinal inflammation (fecal calprotectin [fCal], lipocalin-2 [fLcn2], neopterin), and permeability (plasma lipopolysaccharide [LPS] antibodies, LPS-binding protein) by enzyme immunoassays. Control specimens were obtained from children without CF who had undergone esophagogastroduodenoscopy and had no evidence of gut inflammation.

**Results:**

Twenty-six of 29 participants with CF completed the study. Sixty-nine stools (57 case/12 control) and 76 plasmas (60 case/16 control) were analyzed. LPS antibody had reliable intraindividual stability. fCal, fLcn2, and neopterin were significantly greater in CF than in control samples. fCal was negatively correlated with 3-month interval change (Δ) in weight-for-age z-score, body mass index/weight-for-length z-score, and forced expiratory volume in 1 second. fLcn2 was negatively correlated with FEV1 but not with anthropometrics. No marker correlated with Δbody mass index/weight-for-length z-score or ΔFEV1.

**Conclusions:**

fLcn2 is elevated in people with CF and might predict worse interval pulmonary function. Expanded studies are warranted to test if fLcn2 correlates with changes in additional outcomes.

Suboptimal lean body mass, short stature, and poor nutrient absorption frequently occur in people with cystic fibrosis (PwCF),^1^ and poor nutritional status correlates strongly with diminished pulmonary function and survival.^2^ Well-recognized digestive consequences of cystic fibrosis (CF) are decreased bicarbonate and fluid secretion by the pancreas and proximal intestine and absent/decreased pancreatic enzyme secretion and function. These factors contribute to intestinal mucus accumulation, gut dysmotility, and bacterial dysbiosis.^2^^,^^3^

PwCF have increased gut permeability^4^, ^5^, ^6^, ^7^, ^8^, ^9^ and chronic intestinal inflammation.^10^, ^11^, ^12^ Gut inflammation is associated with poor growth and worse pulmonary outcomes.^10^^,^^12^ However, previous studies of intestinal permeability did not test the relationship of this gut pathobiology to outcomes such as lung function and growth over time. Furthermore, these measures of intestinal permeability were limited by variations in dosing and timing of urine collection, or the use of mannitol (and not rhamnose) as the monosaccharide.^13^^,^^14^ These studies also were performed before the advent of highly effective CF transmembrane conductance regulator (CFTR) modulator therapies that increase duodenal pH toward normal and improve overall nutritional status.^15^ Observational data from human cohorts and experimental data from mice now suggest roles for the gut and its microbial contents in many extraintestinal disorders.^16^, ^17^, ^18^, ^19^ For these reasons, we systematically interrogated gut function and its relation to clinical outcomes in children and adolescents with CF by testing the suitability and repeatability of indicators of gut permeability and inflammation.

Here, we tested a panel of biomarkers of gut inflammation (fecal calprotectin [fCal], lipocalin-2 [fLcn2], and neopterin [fNeo]) and permeability (circulating antibodies to bacterial lipopolysaccharide [LPS], LPS-binding protein [LBP]) (Table I; online available at www.jpeds) to assess the distribution of these markers in PwCF and controls, and to test the hypothesis that intraindividual variation in these values is constrained, reliable, and repeatable over short intervals. The ultimate goal of this work was to determine whether these biomarkers of gut function and pathophysiology correlate with host clinical outcomes, including pulmonary function, growth, and response to CFTR modulator therapy.

## Methods

### Participants

#### Cases

This study was approved by the Human Research Protection Office of Washington University School of Medicine in St Louis (approval number 202107180). Participants were recruited from the St Louis Children’s Hospital CF Care Center. PwCF aged 1-21 years old or their families were approached and informed consent was obtained. Exclusion criteria, aimed at reducing potential confounders, included patients with celiac disease, inflammatory bowel disease, gastroenteritis in the previous 2 weeks, current parenteral nutrition, colostomy, or ileostomy. Participants were enrolled on a rolling basis before their regular quarterly CF center appointments between October 2021 and November 2022. All initial study visits occurred from November 2021 to December 2022, and the last follow-up study visit was in February 2023. This was a convenience sample for a pilot study aimed at supporting sample size calculations for future work involving these biomarkers.

#### Controls

Control subjects were drawn from 2 separate, past studies,^14^^,^^20^ including patients <21 years of age who had undergone upper endoscopy without evidence of suspected chronic inflammation (ie, no evidence of inflammatory bowel disease, eosinophilic esophagitis, or celiac disease). There were 12 stool samples and 16 plasma samples available from a total of 18 control participants.

### Study Methods

Participants with CF were studied at entry and 2 weeks and 3 months later, at which times we obtained stool and blood, and administered questionnaires about gastrointestinal symptoms, such as stool quality, abdominal pain, nausea/vomiting, and heartburn, and about their CF symptoms and quality of life (Cystic Fibrosis Questionnaire–Revised).^21^^,^^22^ The entry and 3-month visits corresponded with protocol quarterly visits to the CF center. The 2-week visit was optional because it was not coordinated with a clinic visit, and for a subset of participants participating in this visit was not practical.

At the entry and 3-month visits, participants had weight and height/length determinations, and age- and sex-adjusted z scores for body mass index (BMI), weight, height/length, and weight-for-length/height were determined using Centers for Disease Control and Prevention growth charts.^23^ Values for standard spirometry, reported as percent predicted forced expiratory volume in 1 second (FEV1) and forced vital capacity using Global Lung Function Initiative predicted equations,^24^ where determined as part of clinical care, were recorded. These tests typically were performed only in participants who were at least 4 years old.^25^

### Data Collection

Each case participant’s height, weight, BMI, weight-for-length z-score, and pulmonary function testing were obtained by chart review and used to determine preenrollment growth and lung function trajectories (Δ variables). Other clinical data obtained via chart review included most recent laboratory tests, including those that reflect pancreatic function (circulating fat-soluble vitamin concentrations and fecal elastase), hepatocellular (transaminases) and biliary (blood gamma-glutamyl transpeptidase and alkaline phosphatase) injury; CFTR genotype; modulator use; time since last pulmonary exacerbation (defined as the interval between cessation of intravenous antibiotics and study assessment); any oral antibiotic use in previous week; all other medications, spanning prescription and over-the-counter drugs, probiotics, supplements, and type of pancreatic enzyme replacement; and current use of enteral feedings.

### Sample Processing

In total, 5 mL of whole blood was collected in ethylenediaminetetraacetic acid tubes, placed on ice, and separated (4°C, 1500*g*, 10 minutes) within an hour of collection. Resulting plasma was aliquoted into 0.5-mL tubes. Stool was produced and collected at home, brought to the visit in a cooler bag with ice packs, and aliquoted into 2-mL tubes. After aliquoting, all plasma and stool samples were frozen (−80°C) until analysis.

### Biomarkers

#### Intestinal Permeability

Surrogate measures of gut permeability consisted of circulating antibodies to *Escherichia coli* anti-core LPS by enzyme immunoassay (EIA) and plasma concentrations of LBP (HK315-02; Hycult Biotech). LBP was performed per the manufacturer’s instructions, with plasma diluted to 1:1000. Measurement of LPS antibody was performed as published,^26^ with plasmas diluted to 1:1000 in 0.5% bovine serum albumin (BSA) in phosphate-buffered saline with 0.05% Tween-20, and repeated with greater or lesser dilutions for samples in which the EIA values were outside the bounds of the standard curve. All assays were performed in duplicate. The EIA uses as antigen the core LPS purified from *E coli* O157:H7 strain 86-24^nalR^ Δ*rfbE* that does not express the O157 side chain, so antibodies detected reflect anti-*E coli* and not anti-*E coli* O157 LPS. The secondary antibody for these EIAs was goat anti-human IgG/F(ab)2-horseradish peroxidase (Millipore Sigma; diluted 1:10 000 in 0.5% BSA in phosphate-buffered saline with 0.05% Tween-20). Results for the anti-LPS antibodies are expressed as EIA units, normalized to a positive control as we have done previously.^26^

#### Intestinal Inflammation

Measures of intestinal inflammation consisted of fCal (HK379-02; Hycult Biotech), fNeo (GWB-286F41; GenWay), and fLcn2 (DY1757; R&D Systems), performed according to the respective manufacturer’s instructions. Stools were diluted to 1:1000, 1:100, and 1:10 000, respectively. For fCal and fLcn2, we performed the first dilution of 1:50 with fecal extraction buffer (0.1 M Tris. HCL [8.0], 0.15 M NaCl, 1 M urea, 10 mM CaCl_2_, 0.1 M citric acid monohydrate, 5 g/L BSA, and 0.25 mM thimerosal) and subsequent dilutions with reagent dilution buffer provided by the respective kits. For fNeo, we performed all dilutions with normal saline. All assays were performed in duplicate. Additional dilutions were performed for out-of-range optical density values until in-range values were obtained.

### Statistical Analysis

Statistical analysis was performed using SAS software, version 9.4 (SAS Institute Inc). All obtained samples were assayed and included in the analyses. Because the data for continuous variables were not normally distributed, we used nonparametric techniques for statistical comparisons and present values as median and IQR. Spearman correlations were used to test relations between outcome variables, and the *P* values compared these correlations to the null hypothesis. To examine inter- and intraindividual variations, linear mixed models were used to include patient ID as a random effect. The variance estimate for Patient ID is the interindividual variance estimate and the variance estimate for residual is the intraindividual variance estimate. The coefficient of variation (CV) was reported. For comparisons between groups, the data were rank transformed before analysis because the residuals were not normally distributed in the raw data. The *P* value was then associated with the linear mixed model with random effect accounting for within-participant correlation. As a sensitivity analysis, we also performed comparisons using the Wilcoxon rank sum test or Kruskal–Wallis test. A 2-tailed *P*-value < .05 was considered statistically significant.

We performed linear mixed modeling with random effects accounting for within-participant correlation to identify associations between biomarkers and clinical outcomes. This modeling technique allowed calculation of a slope estimate to predict change in outcome for each one unit increase for each biomarker.

We performed correlation analyses to identify potential confounders. Potential confounders that were significantly correlated with biomarkers as well as with the clinical outcomes were included in the adjusted model. Linear mixed models with random effect accounting for within-participant correlation and adjusting for one confounder at a time were then generated. Confounders included in these analyses included type of modulator, age, use of oral antibiotics in past week, proton-pump inhibitor use, azithromycin use, and pancreatic insufficiency.

## Results

Of the 29 enrolled participants with CF (cases), 26 completed the study. One participant withdrew before the entry visit because of fear of blood draw, and 2 withdrew after the entry visit because of schedule conflicts (Figure 1; online available at www.jpeds.com). We used stored specimens from 18 control participants from 2 previous studies.^14^^,^^20^ Sixty-nine stool (57 case, 12 control) and 76 blood (60 case, 16 control) samples were collected and analyzed. Characteristics of study participants at each visit and controls are shown in Table II. The 2-week visit occurred 14 (12-14.5) days (median [IQR]) after the entry visit. The 3-month visit occurred 91 (89-105) days (median [IQR]) after the entry visit. All participants (including cases and controls) were non-Hispanic White but one (case), who was multiracial (non-Hispanic White and Black).

**Table II:** Characteristics of study participants at each visit

Characteristics	CF (n = 29)			Controls∗ (n = 18)
	Entry (n = 28)	2-wk visit (n = 11)	12-wk visit (n = 26)	
Age, y	6.9 (2.8-10.9)	3.2 (2.4-9.3)	9.3 (3.0-11.7)	13.3 (11.3-15.9)
Male/female	18 (64)/10 (36)	7 (64)/4 (36)	17 (65)/9 (35)	5 (28)/13 (72)
Distance from CF center,† km	111 (53-217)	53 (51-63)	76 (53-212)	
Modulator(s)	19 (68)	7 (64)	18 (69)	
Elexacaftor/tezacaftor/ivacaftor	11 (39)	3 (27)	12 (46)	
Ivacaftor	3 (11)	2 (18)	3 (12)	
Lumacaftor/ivacaftor	1 (4)	1 (9)	2 (8)	
Tezacaftor/ivacaftor	1 (4)	1 (9)	1 (4)	
Pancreatic insufficiency‡	25 (89)	9 (82)	23 (88)	
Proton-pump inhibitor	20 (71)	9 (82)	17 (65)	
Long-term oral azithromycin	8 (29)	1 (9)	8 (31)	
Weight-for-age z score§	0.12 (−0.45 to 0.67)	0.48 (0.12-1.35)	0.24 (−0.51 to 0.76)	0.255 (−0.6 to 0.69)
Height-for-age z score§	0.01 (−0.65 to 0.47)	0.42 (−0.12 to 0.53)	−0.01 (−0.30 to 0.37)	−0.48 (−1.00 to 0.32)
Weight-for-length/height z score§	0.59 (0.09-0.86)	0.73 (0.30-0.88)	0.23 (−0.12 to 0.8)	n/a
BMI z score§	0.20 (−0.09 to 0.97)	1.05 (0.76-1.61)	0.38 (−0.47 to 1.33)	0.04 (−0.73 to 0.82)
IV antibiotics in past 3 mo	0 (0)	0 (0)	1 (4)	
Oral antibiotics in past week	4 (14)	1 (9)	1 (4)	
Diagnosis of CF liver disease	4 (14)	2 (18)	4 (15)	
Abdominal pain in past month				
None	13 (50)	6 (55)	14 (70)	
Once a week	8 (31)	3 (27)	2 (10)	
2-3 times per week	4 (15)	1 (9)	4 (20)	
4-5 times per week	1 (4)	1 (9)	0 (0)	
Stool provided	25 (89)	10 (91)	22 (85)	12 (75)
Blood provided	28 (100)	10 (91)	22 (85)	16 (89)

*IV*, intravenous; *n/a*, not applicable.

Values expressed as median (IQR) or n (percent).

∗ As published in Holtz et al and Sutton et al.^14^^,^^20^

† Calculated as shortest driving distance from street address of primary residence to St Louis Children’s Hospital in Google Maps in May 2023.

‡ Pancreatic insufficiency defined as those who have low concentrations of fecal elastase, 2 severe mutations, or who are prescribed pancreatic enzyme replacements.

§ As published in Kuczmarski et al.^23^

**Figure 2:**
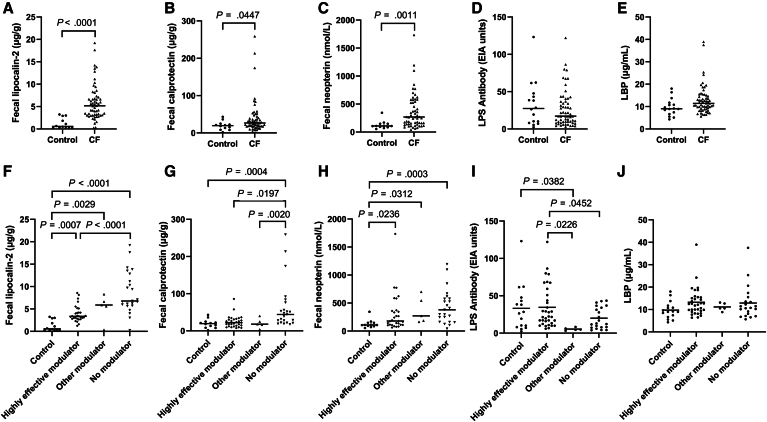
Assessment of biomarkers in control and CF samples *Top row*: **A,** fecal lipocalin-2; **B,** fecal calprotectin; **C,** fecal neopterin; **D,** plasma LPS antibody; **E,** plasma LBP. *Bottom row*: stratified by type of modulator use, **F,** fecal lipocalin-2; **G,** fecal calprotectin; **H,** fecal neopterin; **I,** LPS antibody; **J,** LPS. Other modulator = lumacaftor/ivacaftor, tezacaftor/ivacaftor; highly effective modulator = ivacaftor, elexacaftor/tezacaftor/ivacaftor. The *horizontal line* signifies the median value. The *P*-value was associated with linear mixed model with random effect accounting for within-participant correlation. Two-tailed *P*-values <.05 noted.

The values for these biomarkers in the control and CF groups are shown in Figure 2, with fLcn2, fCal, and fNeo having significantly greater levels in CF compared with control participants. Because the CF group, in the aggregate, was younger than the control group, we performed a sensitivity analysis with age-matched cases >10 years of age; the differences between fNeo and fLcn2 remained significant, but fCal was no longer significantly different between the 2 groups (Table III; online available at www.jpeds.com). As a sensitivity analysis for the *P* values associated with the linear mixed model, we also calculated *P* values using the Wilcoxon rank sum test, and the level of significance remained unchanged (Table III; online available at www.jpeds.com).

For the biomarkers of inflammation that significantly differed between CF and controls (fLcn2, fCal, fNeo), we stratified the CF group into subgroups based on type of modulator use and compared these subgroups with each other, as well as with controls (Figure 2; Table III; online available at www.jpeds.com), and noted that those on highly effective modulators tended to have lower levels of intestinal inflammation than those not on modulators, approaching, but not reaching, that of controls. As a sensitivity analysis for the *P* values associated with the linear mixed model, we also calculated *P* values from the Kruskal–Wallis test, and the level of significance remained unchanged for all biomarkers (Table III; online available at www.jpeds.com).

Correlations between each biomarker and clinical outcomes (growth and pulmonary function) over 3 months are presented in Table IV and suggest that elevations in fLcn2 and fCal are negatively correlated with FEV1% predicted, whereas fCal is also negatively correlated with weight-for-length z-score (WLZ)/body mass index z score (BMIZ) and interval change in weight-for-age z score (WAZ). Given the broad interval at which the 3-month visit occurred, we also looked at correlation of each biomarker with change per day in ΔBMIZ/WLZ, ΔWAZ, and ΔFEV1 to standardize the differing time intervals and did not see a significant change in these associations. After adjusting for age and pancreatic insufficiency (separately, with each model adjusting for one confounder at a time), the association between BMIZ/WLZ and the biomarkers did not significantly change (Table V; online available at www.jpeds.com). After adjusting for use of oral antibiotics in the past week, LPS antibody became significantly positively correlated with FEV1% predicted (Table V; online available at www.jpeds.com). PPI use and azithromycin use did not confound these models because they were not significantly correlated with the outcomes or biomarkers.

**Table IV:** Association of biomarkers with clinical outcomes, including pulmonary function (FEV1% predicted, interval change [Δ] in FEV1% predicted∗) and growth (interval change in BMIZ/WLZ, interval change in WAZ, and BMIZ/WLZ)

Biomarkers	FEV1% predicted		ΔFEV1% predicted		ΔBMIZ/WLZ		ΔWAZ		BMIZ/WLZ	
	Estimate	*P* value	Estimate	*P* value	Estimate	*P* value	Estimate	*P* value	Estimate	*P* value
fLcn2, μg/g	**−1.253**	**.006**	−0.2438	.5157	0.001	.967	−0.007	.480	−0.019	.442
fCal, μg/g	**−0.102**	**.029**	0.01112	.7308	−0.002	.242	**−0.002**	**.032**	**−0.009**	**<.001**
fNeo, nmol/L	−0.003	.587	0.001399	.7137	0.000	.579	0.000	.520	0.000	.809
LPS antibody, EIA units	−0.041	.628	−0.02808	.5434	0.002	.588	0.000	.796	0.001	.794
LBP, μg/g	0.253	.592	−0.1207	.6892	−0.006	.545	−0.010	.084	**−0.018**	**.044**

Interval change is considered the change over the preceding ∼3 months since last CF visit. The estimates predict the change in the clinical outcome if the biomarker increases by 1 unit and were generated using a linear mixed model with random effect accounting for within-participant correlation. Estimates with *P* value < .05 are bolded.

∗ As published in Cooper et al.^24^

fLcn2 positively correlated with both fCal (Spearman rho = 0.570, *P* < .001) and fNeo (Spearman rho = 0.408, *P* = .0005). LPS antibody also positively correlated with LBP (Spearman rho = 0.392, *P* = .001). The other biomarkers did not significantly correlate with each other.

To measure the intraindividual variation of biomarkers over intervals, we determined Spearman correlation coefficients for values obtained at each visit. LPS antibody significantly correlated between each visit, although the correlation between the entry and 2-week visits (Spearman rho = 0.830, *P* = .003) was less than between the 2-week and 3-month visits (Spearman rho = 0.933, *P* = .000); this was unexpected, given that the entry and 2-week visits are closer in time. fLcn2 did have a greater correlation for the entry and 2-week visits (Spearman rho = 0.479, *P* = .162) compared with the 2-week and 3-month visits (Spearman rho = 0.333, *P* = .381) or the entry and 3-month visits (Spearman rho = 0.368, *P* = .092); however, these correlations were not statistically significant.

To compare inter- and intraindividual variability, the CVs were calculated, including all visits in the analysis, and separately, including only the entry and 2-week visits, because these were closer in time and thus may have a lesser CV (Table VI). The intraindividual CV was less than the interindividual CV for LPS antibody (Table VI).

**Table VI:** Inter- and intraindividual variability for each biomarker

Biomarker	Visits included	Mean (SE)	VAR_inter_	VAR_intra_	CV_inter_, %	CV_intra_, %
fLcn2, μg/g	1, 2, 3	6.0 (0.6)	4.8	11.9	36.4	57.0
	1, 2	4.9 (0.9)	6.9	3.9	53.4	40.1
fCal, μg/g	1, 2, 3	40.9 (8.1)	1099.8	1124.7	81.1	82.0
	1, 2	30.2 (4.6)	55.7	327.5	24.7	59.9
fNeo, nmol/L	1, 2, 3	370.0 (56.0)	53 124.3	54 393.3	62.3	63.0
	1, 2	341.9 (57.9)	15 960.4	39 467.9	36.9	58.1
LPS antibody, EIA units	1, 2, 3	24.8 (4.2)	437.8	124.4	84.4	45.0
	1, 2	31.4 (9.3)	846.9	183.6	92.7	43.2
LBP, μg/mL	1, 2, 3	12.8 (0.9)	7.1	34.1	20.8	45.7
	1, 2	14.5 (1.6)	2.4	49.4	10.6	48.5

*CV*_*inter*_, interindividual CV; *CV*_*intra*_, intraindividual CV; *VAR*_*inter*_, interindividual variance estimate; *VAR*_*intra*_, intraindividual variance estimate.

## Discussion

These data demonstrate fLcn2 concentrations in PwCF that are strongly associated with case status, vary according to category of modulator use, and negatively correlate with pulmonary function. The other fecal biomarkers (fCal and fNeo) corroborate that intestinal inflammation is associated with case status and, to a lesser degree, modulator status. These data are also consistent with previous studies that demonstrated a decrease in intestinal inflammation, as measured by fCal, with CFTR modulator therapies.^27^^,^^28^ The use of fLcn2 in future studies in CF is therefore promising and should be explored, in particular to interrogate correlation with growth.

The potential etiology of intestinal inflammation in PwCF is broad. There is evidence of gut dysbiosis, likely related to defective mucus accumulation, impaired gut motility, and frequent antibiotic use.^2^^,^^3^ Intestinal inflammation was reduced with the administration of probiotics,^29^ indicating a possible link between gut dysbiosis and inflammation, which is also supported by the negative correlation between fCal and *Akkermansia*, which has anti-inflammatory properties.^30^ Furthermore, dietary intake with the high-energy, high-fat diet in PwCF may play a role in inflammation, as PwCF have a lower intake of whole grains, starch, and fiber and greater intake of saturated and trans fats.^30^ Fiber and whole grains can have anti-inflammatory properties, and specifically whole grain intake was negatively correlated with fCal in a CF cohort.^30^

Because lipocalin-2 is expressed by neutrophils and by epithelial cells in gastrointestinal, respiratory, and urogenital systems, fLcn2 could reflect intestinal cell turnover in addition to inflammation.^31^ Indeed, fLcn2 is a sensitive marker for both low-grade/subclinical and more robust inflammation in mice, and its reported stability at room temperature for at least 24 hours makes it further convenient for testing.^32^ In contrast to calprotectin, the expression of lipocalin-2 in biopsies from the ileum and rectum of histologically healed patients with inflammatory bowel diseases remained elevated compared with controls, showing its sensitivity in states of lower-grade inflammation.^33^ In biopsy-proven celiac disease compared with controls, there was a significant elevation of fLcn2 but not fCal, indicating the possible increase in sensitivity of fLcn2 compared with fCal for small intestinal pathology or enteropathy.^20^ In addition, PwCF have a greater risk of colorectal cancer; Dayama et al demonstrated that the gene that encodes for lipocalin-2 is one of the most upregulated genes in their participants with CF^34^ and is known to be overexpressed in colorectal cancer. These data further support the potential for fLcn2 as a biomarker for gut dysfunction in CF. However, additional studies are needed to examine target stability (at room temperature beyond 24 hours and after freezing), reliability of the assays, and intra-day variation. Future studies should also assess blood, sputum, and urine lipocalin-2 concurrently, given its expression in neutrophils and the various epithelial cells, to ensure that the fecal levels represent a true gut epithelial marker and not swallowed sputum.

Although some markers of intestinal inflammation correlated with overall FEV1 and BMIZ/WLZ, similar to what has been shown in previous studies such as Dhaliwal et al^10^ and Parisi et al,^12^ none correlated with ΔFEV1 or ΔBMIZ/WLZ, and only fCal correlated with ΔWAZ. The interval change was calculated over the preceding 3 months; 3 months may not be sufficient to see downstream effects of gut inflammation reduction. In addition, FEV1 was normal in most of these participants, so small changes in FEV1 might not have been clinically meaningful. There are variations in how FEV1 change can be measured (absolute or relative change), and pediatric populations may need more sensitive indicators of early progression of lung disease for future studies, given that their FEV1 may be normal until adulthood.^35^ Similarly, anthropometry can vary based on technique and time of day, so the change in one measurement is not as useful in the overall trend of multiple measurements,^36^ although our CF center does have standardized methods with which they perform anthropometry to minimize these variations. Although this study did not demonstrate correlation of these biomarkers with interval changes, we believe that these gut biomarkers are worthy of further inquiry over longer intervals to more definitively confirm or refute their predictive values in clinical outcomes, especially as it is difficult to demonstrate linear growth over only 3 months.

Our data provide some guidance for future sampling strategies. The intraindividual biomarker variability was not completely unexpected, as fCal is known to have considerable intra-day change in ulcerative colitis.^37^ The intraindividual variability for visits closer in time was not consistently less than the intraindividual variability between all the visits nor the interindividual variability. In addition, visits closer in time did not correlate more strongly than the other visits except for fLcn2, and even this correlation was not statistically significant. LPS antibody had the greatest intraindividual stability; this biomarker, even though it did not correlate with clinical outcomes, strongly correlated between each visit and had lesser intraindividual than interindividual variability. The variability in our data suggest that a 2-week interval may be long enough to have significant changes in gut permeability and inflammation, possibly because of illnesses, diet, medications, or exposures. We also acknowledge that host biology that underlies the expression of these markers could be prone to saccadic bursts, in which case values integrated over time might be more reliable. Regardless, these results reinforce that gut dysfunction, which seemingly responds to modulator therapy, exists in PwCF and offer some mechanistic insight in this population. It will be important to learn more about the pathobiology of marker expression, before constructing case use scenarios in which they can be employed clinically.

The similarity between CF and controls for LPS antibody and LBP was unexpected. Because previous studies have shown increased intestinal permeability in PwCF as measured by dual-sugar permeability testing,^1^, ^4^, ^5^, ^6^, ^7^, ^8^, ^9^ we hypothesized that these surrogate measures of gut permeability would be elevated in our case participants. Our data did not support this hypothesis. The literature offers mixed guidance on the value of circulating LPS antibody and LBP concentrations as markers of intestinal permeability. Some studies, involving cohorts in Crohn’s disease, cirrhosis, and obesity, have not found a correlation between lactulose/mannitol ratio in dual sugar absorption testing and LBP,^38^, ^39^, ^40^ whereas others, also involving cohorts with obesity, have found association independent of age, sex, and BMI.^41^ In addition, these markers can be affected by factors such as weight, dietary fat content, age, and frequent infections^38^^,^^42^; none of these were adequately addressed in this pilot study.

We acknowledge several study limitations. As discussed previously, the assessment of intestinal permeability could have been strengthened by concurrent measurement of dual sugar permeability testing. The sample size was small, with many missing values, especially for the 2-week measurements, making it difficult to assess true inter- and intraindividual variability, especially because the data were not normally distributed. Since this was an exploratory study with a limited sample size, no adjustments in the analysis were made for multiple comparisons. In addition, the participants may not be representative of all PwCF, given selection bias; those choosing to participate also may be the ones who have easier transportation to the center for CF visits and/or are more able to adhere to their medications and treatments. Participants frequently traveled from far distances with their stool samples, and despite the use of ice packs, we could not control preprocessing variables. The controls were not age-matched, which affected some of the comparisons between CF and controls. In particular, elevation of fCal in PwCF was no longer significantly different when comparing only age-matched PwCF with controls. The controls also produced only one blood or stool sample and did not include complete data on medication use. Also, we had incomplete data on nonsteroidal anti-inflammatory drugs in our participants, which are interventions that can affect intestinal permeability.^43^ Although there is a well-known relationship between fCal and age,^44^ our analyses included age as a potential confounder.

In summary, our data demonstrate a high degree of variability among putative fecal and plasma biomarkers of gut permeability and inflammation, with circulating LPS antibody being the most stable. Despite this variability, fLcn2 showed promise as a potential biomarker in CF that is negatively correlated with pulmonary function. There remains a pressing need to study gut function and its effect on the course of CF, and rigorous validation of measures of intestinal permeability and inflammation in PwCF will be required for use of these biomarkers in future studies.

## Data Statement

Data sharing statement available at www.jpeds.com.

## Declaration of Competing Interest

Supported by the 10.13039/100000897Cystic Fibrosis Foundation (DUCKWO21B0, 003630D122 to L.D.); and the 10.13039/100000865Bill & Melinda Gates Foundation (OPP1066153 to L.H.). The Cystic Fibrosis Foundation and Bill & Melinda Gates Foundation were not involved in the study design, collection, analysis, and interpretation of data; the writing of the report; or the decision to submit for publication. These data have been accepted in abstract form to the North American Cystic Fibrosis Conference 2023, and the abstract was published in an online supplement to *The Journal of Cystic Fibrosis* in October 2023 and the work was presented at in poster form. P.T. is a holder of equity in, a consultant to, and a member of the Scientific Advisory Board of MediBeacon Inc, which is developing technology to noninvasively measure intestinal permeability in humans. He is also a coinventor on patents assigned to MediBeacon, which might earn royalties if the technology is commercialized. The other authors have no conflicts of interest to disclose.

## Appendix

STROBE-checklist

Tables I, III, V

Figure 1 Inclusion and exclusion of participants and sample collection. *CF*, cystic fibrosis.

Data Statement
