# Supplementary material for: Quantification of Enteric Dysfunction in Cystic Fibrosis: Inter- and Intraindividual Variability
Source: J Pediatr. 2024 Feb;265:113800. doi: 10.1016/j.jpeds.2023.113800 (PMC10869934; doi:10.1016/j.jpeds.2023.113800)
Supplement: Tables I, III, V [file mmc2.docx]

**Table 1**: Hypothesized immune pathways and abbreviations for each biomarker

| **Biomarker** | **Abbreviation** | **Immune pathway** |
| --- | --- | --- |
| Fecal calprotectin | fCal | Produced by activated neutrophils, reflects innate immune system^(1)^. |
| Fecal lipocalin-2 | fLcn2 | Found in neutrophils, so also reflects innate immune system. Also produced by intestinal, urogenital, and respiratory epithelial cells^(2)^. |
| Fecal neopterin | fNeo | Produced by macrophages and dendritic cells in response to INF-γ, which is stimulated by Th1 helper T-cells, reflecting cell-mediated inflammation^(3)^. |
| Lipopolysaccharide antibody | LPS antibody | LPS is produced by gram-negative bacteria and is an activator of the innate immune system. LPS antibody binds to LPS and indicates exposure^(4)^. |
| Lipopolysaccharide-binding protein | LBP | Induced by LPS and binds to LPS during the innate immune system^(4)^. |

**Table 3**: Comparison of biomarkers in control versus age-matched CF (where CF patient age >10 years), all CF participants, and CF stratified by type of modulator use. Values shown as median [interquartile range]. Other modulator=lumacaftor/ivacaftor, tezacaftor/ivacaftor; highly effective modulator= ivacaftor for indicated ivacaftor-responsive mutation, elexacaftor/tezacaftor/ivacaftor

| **Variables** | **Control** | **Age-matched CF (>10 years)** | | | **All CF** | | | **CF (type of modulator)** | | | | |
| --- | --- | --- | --- | --- | --- | --- | --- | --- | --- | --- | --- | --- |
|  |  | **Age-matched CF** | **P-value^1^** | **P-value^2^** | **All CF** | **P-value^1^** | **P-value^2^** | **No Modulator** | **Other Modulator** | **Highly Effective Modulator** | **P-value^1^** | **P-value^3^** |
| Age (years) | 13.3 [11.3,15.9] | 13.5 [10.8,17.9] |  |  | 6.9  [2.8,10.9] |  |  | 2.1  [1.6,4.2] | 6.8  [2.8,10.7] | 10.0  [8.8,15.1] |  |  |
| fLcn2 (μg/g) | 0.6  [0.3,2.4] | 4.2  [3.1,7.3] | <.0001 | 0.0008 | 5.2  [3.1,7.1] | <.0001 | <.0001 | 6.8  [5.7,12.8] | 5.9  [5.6,6.6] | 3.4  [2.8,4.2] | <.0001 | <.0001 |
| fCal (μg/g) | 19.6 [10.8,23.6] | 23.5 [15.0,34.6] | 0.3083 | 0.2911 | 26.7 [18.1,40.0] | 0.0447 | 0.0442 | 44.0 [22.2,76.2] | 18.0 [16.7,24.9] | 22.2 [14.0,30.9] | 0.0017 | 0.0005 |
| fNeo (nmol/L) | 108.4 [89.0,147.2] | 265.3 [142.5,455.4] | 0.0036 | 0.0057 | 269.1 [144.3,543.5] | 0.0011 | 0.0013 | 378.4 [161.7,578.3] | 270.0 [185.5,543.5] | 178.9 [112.4,359.0] | 0.0033 | 0.0016 |
| LPS antibody (EIA units) | 27.5 [9.0,45.4] | 19.9 [9.9,34.3] | 0.6908 | 0.9151 | 17.1 [9.7,36.8] | 0.4025 | 0.5469 | 12.9 [7.8,34.0] | 4.8  [4.4,6.2] | 22.0 [13.3,50.9] | 0.0434 | 0.0031 |
| LBP (μg/mL) | 9.0  [7.1,11.3] | 10.1 [8.9,13.7] | 0.1504 | 0.1162 | 11.5 [9.0,14.8] | 0.0919 | 0.0419 | 10.7 [8.1,13.6] | 10.5 [10.5,12.7] | 12.3 [9.4,15.4] | 0.0919 | 0.1837 |

fLcn2=fecal lipocalin-2, fCal=fecal calprotectin, fNeo=fecal neopterin, LPS antibody=lipopolysaccharide antibody, LBP=lipopolysaccharide binding protein

^1^ two-tailed p-value associated with linear mixed model with random effect accounting for within-participant correlation

^2^ two-tailed p-value associated with Wilcoxon rank sum test which was performed as sensitivity analysis

^3^ two-tailed p-value associated with Kruskal-Wallis test which was performed as sensitivity analysis

**Table 5:** The association between biomarkers and clinical outcomes (FEV1% predicted^1^, interval change in body mass index or weight-for-length z-score (BMIZ/WLZ), interval change in weight-for-age z-score (WAZ), and BMIZ/WLZ adjusted for confounders. Only confounders that were significantly correlated with the biomarkers and the clinical outcomes were included in these adjusted models. Each model adjusts for one confounder at a time. The estimate predicts the change in the outcome if the biomarker increases by 1 unit.

|  | **Biomarker estimate** | | | **Confounders** | |
| --- | --- | --- | --- | --- | --- |
| **Clinical outcomes** | **Biomarkers** | **Estimate** | **P-value^2^** | **Confounders** | **P-value^3^** |
| BMIZ/WLZ | fLcn2 | -0.03615 | 0.1526 | Age | 0.0057 |
|  | **fCal** | **-0.00879** | **<.0001** | Age | 0.0186 |
|  | fNeo | 0.000070 | 0.8375 | Age | 0.0143 |
|  | LPS antibody | 0.002393 | 0.5949 | Age | 0.1427 |
|  | **LBP** | **-0.01800** | **0.0431** | Age | 0.1597 |
| Change in BMIZ/WLZ | LBP | -0.00471 | 0.6071 | Type of Modulator | 0.0474 |
| BMIZ/WLZ | **fCal** | **-0.00870** | **<.0001** | Pancreatic Insufficiency | 0.0955 |
| FEV1 % Predicted | **LPS** | **0.7058** | **<.0001** | Oral antibiotics in past week | 0.0393 |

fLcn2=fecal lipocalin-2, fCal=fecal calprotectin, fNeo=fecal neopterin, LPS antibody=antibody to bacterial lipopolysaccharide antibody, LBP=lipopolysaccharide binding protein

^1^(5)

^2^p-value for the adjusted model showing the association between the biomarker and clinical outcome. Significant associates (p < 0.05) are **bolded.**

^3^The p-value for the association between the confounder and clinical outcome adjusted for the influence of the biomarker

References for online tables:

1. Zollner A, Schmiderer A, Reider SJ, Oberhuber G, Pfister A, Texler B, et al. Faecal Biomarkers in Inflammatory Bowel Diseases: Calprotectin Versus Lipocalin-2-a Comparative Study. J Crohns Colitis. 2021;15(1):43-54.

2. Chassaing B, Srinivasan G, Delgado MA, Young AN, Gewirtz AT, Vijay-Kumar M. Fecal lipocalin 2, a sensitive and broadly dynamic non-invasive biomarker for intestinal inflammation. PLoS One. 2012;7(9):e44328.

3. Kosek M, Haque R, Lima A, Babji S, Shrestha S, Qureshi S, et al. Fecal markers of intestinal inflammation and permeability associated with the subsequent acquisition of linear growth deficits in infants. Am J Trop Med Hyg. 2013;88(2):390-6.

4. Fotis L, Shaikh N, Baszis KW, Samson CM, Lev-Tzion R, French AR, et al. Serologic Evidence of Gut-driven Systemic Inflammation in Juvenile Idiopathic Arthritis. J Rheumatol. 2017;44(11):1624-31.

5. Cooper BG, Stocks J, Hall GL, Culver B, Steenbruggen I, Carter KW, et al. The Global Lung Function Initiative (GLI) Network: bringing the world's respiratory reference values together. Breathe (Sheff). 2017;13(3):e56-e64.
