# Supplementary figures and images for: Quantification of Enteric Dysfunction in Cystic Fibrosis: Inter- and Intraindividual Variability
Source: J Pediatr. 2024 Feb;265:113800. doi: 10.1016/j.jpeds.2023.113800 (PMC10869934; doi:10.1016/j.jpeds.2023.113800)

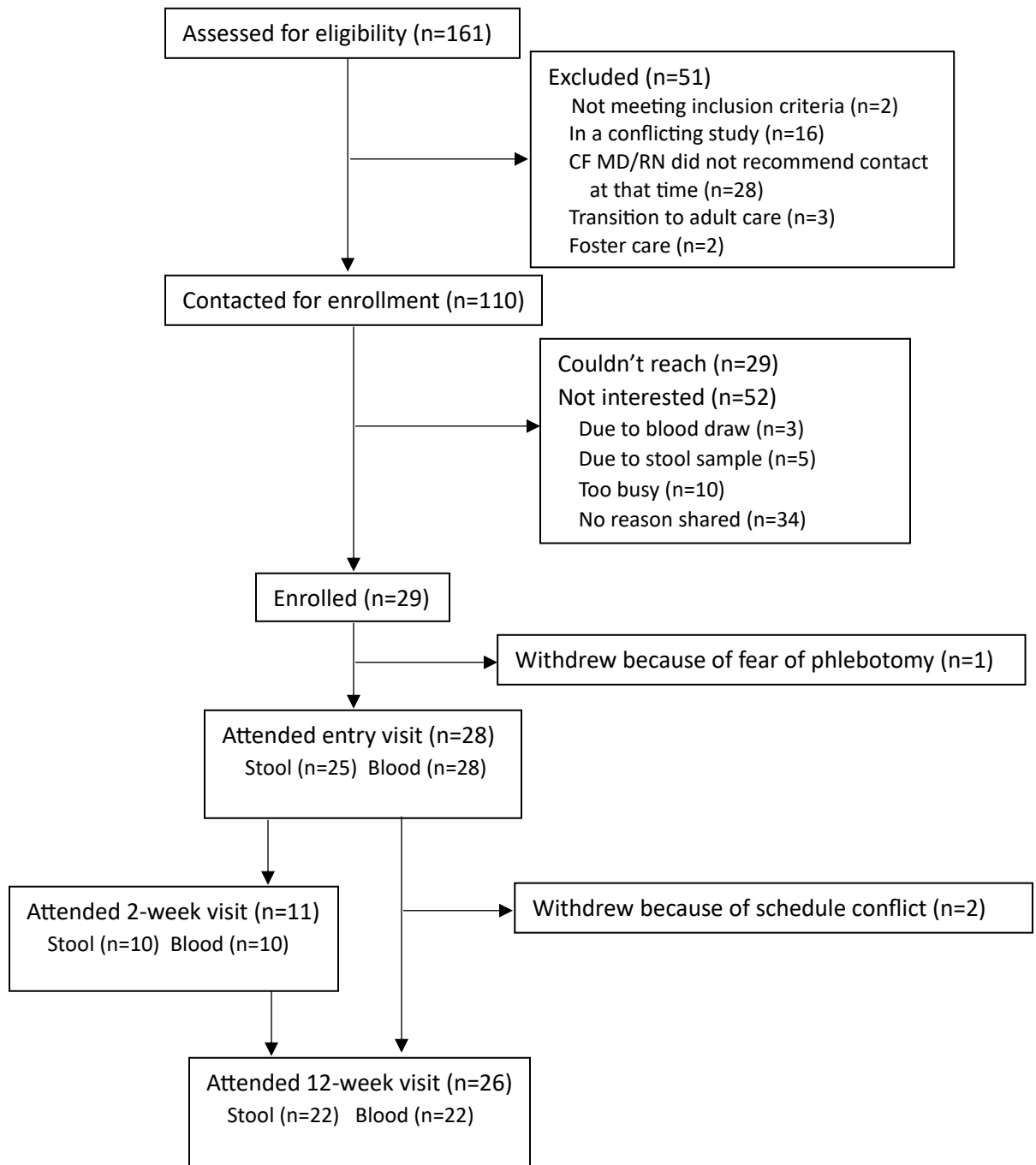

Supplement: Figure 1 — Inclusion and exclusion of participants and sample collection. CF, cystic fibrosis. [file mmc3.pdf]
