# Supplementary material for: Quantification of Enteric Dysfunction in Cystic Fibrosis: Inter- and Intraindividual Variability
Source: J Pediatr. 2024 Feb;265:113800. doi: 10.1016/j.jpeds.2023.113800 (PMC10869934; doi:10.1016/j.jpeds.2023.113800)
Supplement: Data Statement [file mmc4.docx]

Data sharing statement: Data collected during the study, after deidentification, may be shared with qualified researchers upon request to L.A.D. ([albertl@wustl.edu](mailto:albertl@wustl.edu)) and execution of appropriate institutional data sharing agreement. Additional study documents that will be shared include: GI symptom questionnaire.
